# P-Cresyl Sulfate Is a Valuable Predictor of Clinical Outcomes in Pre-ESRD Patients

**DOI:** 10.1155/2014/526932

**Published:** 2014-01-29

**Authors:** Cheng-Jui Lin, Chi-Feng Pan, Chih-Kuang Chuang, Fang-Ju Sun, Duen-Jen Wang, Han-Hsiang Chen, Hsuan-Liang Liu, Chih-Jen Wu

**Affiliations:** ^1^Division of Nephrology, Department of Internal Medicine, Mackay Memorial Hospital, 92 Chung San North Road, 10449 Taipei, Taiwan; ^2^Department of Medical Research, Mackay Memorial Hospital, 10449 Taipei, Taiwan; ^3^Department of Laboratory Medicine, Mackay Memorial Hospital, 10449 Taipei, Taiwan; ^4^Mackay Medicine, Nursing and Management College, 10449 Taipei, Taiwan; ^5^Institute of Biotechnology, National Taipei University of Technology, 10608 Taipei, Taiwan; ^6^Division of Genetics and Metabolism, Department of Medical Research, Mackay Memorial Hospital, 10449 Taipei, Taiwan; ^7^College of Medicine, Fu-Jen Catholic University, 24205 New Taipei City, Taiwan; ^8^Graduate Institute of Medical Science, Taipei Medical University, 11043 Taipei, Taiwan; ^9^Mackay Medical College, 25245 New Taipei City, Taiwan

## Abstract

*Background/Aims*. Previous studies have reported p-cresyl sulfate (PCS) was related to endothelial dysfunction and adverse clinical effect. We investigate the adverse effects of PCS on clinical outcomes in a chronic kidney disease (CKD) cohort study. *Methods*. 72 predialysis patients were enrolled from a single medical center. Serum biochemistry data and PCS were measured. The clinical outcomes including cardiovascular event, all-cause mortality, and dialysis event were recorded during a 3-year follow-up. *Results*. After adjusting other independent variables, multivariate Cox regression analysis showed age (HR: 1.12, *P* = 0.01), cardiovascular disease history (HR: 6.28, *P* = 0.02), and PCS (HR: 1.12, *P* = 0.02) were independently associated with cardiovascular event; age (HR: 0.91, *P* < 0.01), serum albumin (HR: 0.03, *P* < 0.01), and PCS level (HR: 1.17, *P* < 0.01) reached significant correlation with dialysis event. Kaplan-Meier analysis revealed that patients with higher serum p-cresyl sulfate (>6 mg/L) were significantly associated with cardiovascular and dialysis event (log rank *P* = 0.03, log rank *P* < 0.01, resp.). *Conclusion*. Our study shows serum PCS could be a valuable marker in predicting cardiovascular event and renal function progression in CKD patients without dialysis.

## 1. Introduction

Cardiovascular disease is still the main leading cause that resulted in morbidity and mortality in patients with chronic kidney disease (CKD) [1–3]. This high mortality and its underlying causes among CKD patients are a crucial issue. A broad range of traditional risk factors could not fully explain the high risk of mortality in such population [4]. Thus, recent studies have demonstrated that nontraditional risk factors including uremic toxins may play a role in the development of cardiovascular disease in CKD [5–8].

Uremic solutes are accumulated as renal clearance rate declined. Most uremic toxins can be removed by dialysis except protein-bound uremic toxins, due to its higher affinity for serum protein [9]. P-cresyl sulfate (PCS), one kind of protein-bound uremic toxins, has been reported not only to reduce endothelial proliferation but also to inhibit endothelial repair mechanisms [10, 11]. In addition, an increasing evidence suggests that PCS is a valuable predictor of cardiovascular events [12], infection event [13] and all-cause mortality event in hemodialysis patients [14]. However, there is also a significant association of serum PCS with vascular disease in CKD patients. Our recent study also indicated that PCS levels had strong correlation with vascular access dysfunction in patients on maintenance hemodialysis [15].

From these reports, PCS seems a novel and important surrogate in CKD patients. However, its clinical toxic effect still needs to be verified by more studies. Thus, in this study, we further investigated the effect of PCS on clinical outcomes including kidney function progression, cardiovascular event, and all-cause mortality in a pre-ESRD cohort.

## 2. Subjects and Methods 

Seventy-two patients with CKD3–5 were recruited in this study from January to April 2008 in a medical centre. Patients who met the following criteria with acute infection and cardiovascular events in the past 3 months, with malignancy, or those younger than 18 years were excluded. The etiology of CKD in the study patients included cGN, diabetic nephropathy, polycystic kidney disease, or lupus nephritis. Patient characteristics and biochemical parameters were recorded and measured. Our study was performed in accordance with the principles of the Declaration of Helsinki and approved by the Ethics Committee of the Mackay Memorial Hospital. Informed consent was obtained from all patients.

Biochemistry data including the following tests were performed: blood urea nitrogen (BUN, md/dL), creatinine (Cr, mg/dL), hemoglobin (Hb, g/dL), hematocrit (Hct, %), calcium (Ca, mg/dL), phosphate (P, mg/dL), intact-parathyroid hormone (i-PTH, pg/mL), albumin (g/dL), and p-cresyl sulfate (mg/L). Serum albumin levels were determined by bromocresol green method.

Serum PCS were analyzed with LC-MS/MS (4000 QTRAP, USA). Briefly, serum samples were prepared and deproteinized by heat denaturation. HPLC was performed at room temperature using a dC18 column (3.0 × 50 mm, Atlantis, Waters). The buffers used were (A) 0.1% formic acid and (B) 1 mM NH_4_OAc + 0.1% formic acid in 100% acetonitrile. The flow rate was 0.6 mL/min with a 3.5 min gradient cycling from 90% A/10% B to 10% A/90% B. Under these conditions, PCS was eluted at 2.73 min. Standard curves for PCS were set at 1, 5, 10, 50, 250, 500, and 1000 *μ*g/L, and they correlated with the serum samples with average *r* ^2^ values of 0.996 ± 0.003. These samples were diluted if PCS concentration exceeded standard curve. Quantitative results were obtained and calculated in terms of their concentrations (mg/L). The sensitivity of this assay was 1 *μ*g/L for PCS.

Our patients were followed up for 3 years until May 31, 2011. During study period, clinical outcomes including cardiovascular events, all-cause mortality, and dialysis event were reviewed by 1 independent physician (Pan CF), who was blinded for study. The medical charts were reviewed for all dialysis, and for surgeries due to nephrologic, cardiologic, and vascular defects. The cardiovascular event was defined as patients with any one of following events including cardiovascular events including death from cardiac causes, myocardial ischemia, nonfatal myocardial infarction, ischemic stroke, or new onset of peripheral vascular disease, whichever developed first. Only one event of cardiovascular event per subject was included in the analysis. Deaths were accurately recorded and the cause of death were categorized as cardiovascular, infectious, or other. Only patients who met the criteria of starting long-term dialysis including hemodialysis or peritoneal dialysis were recorded as having dialysis events in this study.

The demographic data were expressed as the mean ± standard deviation (SD). Mann-Whitney *U* test was applied for the comparison between two groups divided by a medium PCS level (PCS, ≧6.0 mg/L and <6.0 mg/L) in CKD patients. Cox regression model was used to analyze the relationship between independent variables and clinical outcomes including cardiovascular event, dialysis event and all-cause mortality. All variables with a statistically significant *P* value in the univariate analysis were included in multivariate analysis. The Kaplan-Meier method (factors were compared using the log-rank test) was used to estimate cumulative event free rate of time to first cardiovascular event, time to first dialysis event, and overall mortality in CKD patients with PCS level above and below the median (6.0 mg/L). A value of *P* less than 0.05 was considered statistically significant. All statistical analyses were conducted by using the SPSS version 17.0 software program (SPSS, Chicago, IL).

## 3. Results

72 stable patients with CKD stages 3, 4, and 5 (34.8%, 32.4% and 32.8%, resp.) were recruited in this study. The mean age of patients was 60.6 ± 9.7 years old and this research included 36 males (50%) and 36 females (50%). Patient's demographics and biochemistry are shown in Table 1. 23 patients had diabetes mellitus (31.9%), 31 patients had hypertension (43.1%), and 11 patients had cardiovascular disease (15.3%). All patients were divided into two groups based on median PCS level (6.0 mg/L) (Table 2). Our results revealed that patients with higher serum PCS had significantly lower Hb, Hct, estimated GFR and higher BUN, Cr, and i-PTH. There was no difference on albumin, calcium, and phosphate levels.

At the end of study, 18 out of 72 patients were recorded as experiencing a new cardiovascular event. Only 6 patients died (4 from cardiovascular causes and 2 from infectious disease). In addition, 16 patients started to undergo regular dialysis due to deterioration of renal function including 13 hemodialysis and 3 peritoneal dialysis during 3-year follow-up.

Table 3 revealed the Cox regression analysis results of independent variables on specific clinical outcomes including cardiovascular event, all-cause mortality, and dialysis event. For cardiovascular event, age, CAD, BUN, eGFR, calcium, phosphate, i-PTH, and PCS were significantly associated with cardiovascular event in the univariate Cox regression analysis. After adjusting confounding factors, only age (HR: 1.12, *P* = 0.01), CAD (HR: 6.28, *P* = 0.02), and PCS (HR: 1.12, *P* = 0.02) had reached significance in the multivariate analysis. In addition, age, BUN, Cr, eGFR, albumin, phosphate, i-PTH, and PCS were found independently to relate to dialysis event in the univariate analysis. It showed only age (HR: 0.91, *P* < 0.01), albumin (HR: 0.03, *P* < 0.01), and PCS (HR: 1.17, *P* < 0.01) reached significant association with this event finally. However, there was no association between independent variables and all-cause mortality.

**Figure 1:**
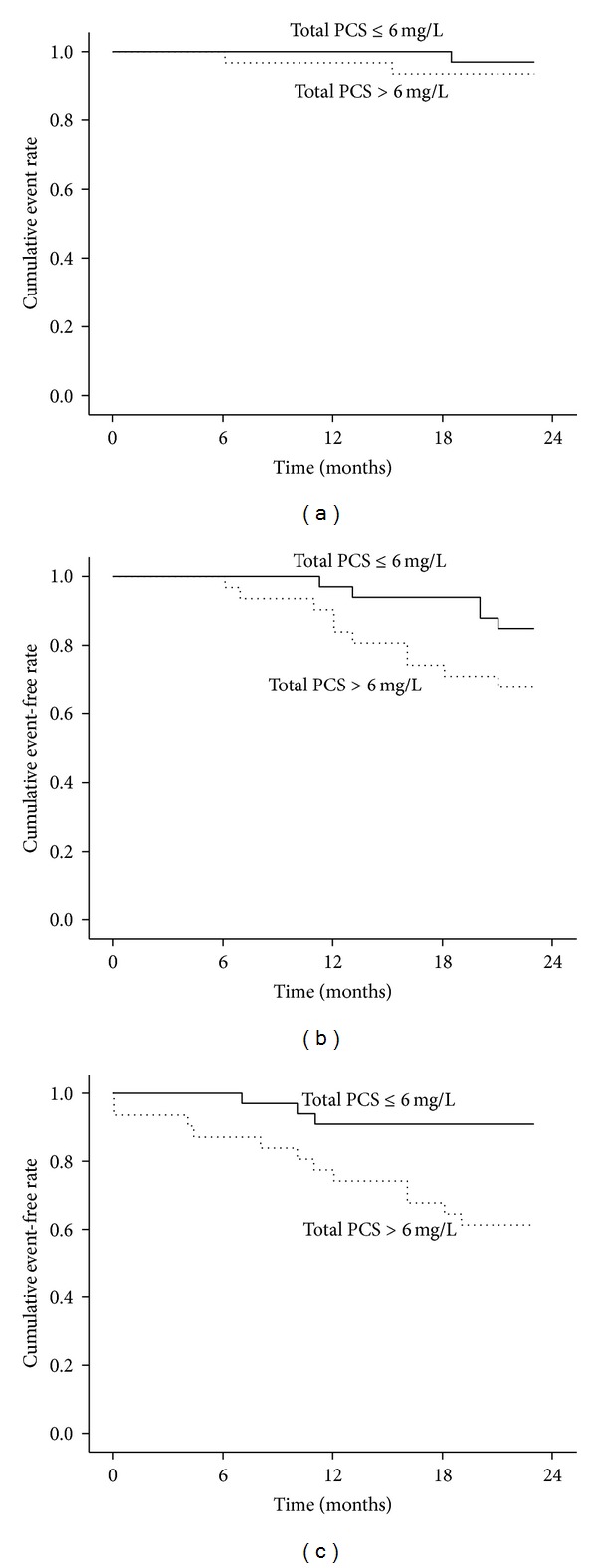
Kaplan-Meier curves of time to first clinical events. Patients were divided into two groups (>6.0 mg/L and ≦6.0 mg/L) by medium level of p-cresyl sulfate. (a) All-cause mortality, log rank *P* = 0.26, (b) cardiovascular event, log rank *P* = 0.03, and (c) dialysis event, log rank *P* < 0.01.

Kaplan-Meier curves of time to the first clinical events were showed in Figure 1. Patients were divided into two groups by median PCS levels (>6.0 mg/L and ≦6.0 mg/L). Patients with higher PCS level were strongly associated with higher rate of a cardiovascular event and dialysis event than those with lower PCS levels during 3-year follow-up (log rank *P* = 0.03, *P* < 0.01, resp.) (Figures 1(b) and 1(c)). However, only 6 patients died at the end of the study. Statistical analysis showed no significant difference for PCS level on all-cause mortality in this CKD cohort (log rank *P* = 0.26) (Figure 1(a)).

## 4. Discussion

Our study showed that serum PCS level was significantly associated with cardiovascular and dialysis events in a predialysis CKD cohort during a 3-year follow-up. From this result, we suggested PCS levels may be an alternative surrogate in prediction of cardiovascular disease and kidney function deterioration.

It is well known that CKD is independently associated with endothelial dysfunction [16], which plays a vital role in the development of cardiovascular diseases and is the main cause of mortality in CKD patients [17]. Thus, it is not surprising that cardiovascular disease remained the most important cause of morbidity and mortality in patients with predialysis and dialysis patients [1–3]. Some traditional and nontraditional risk factors have been reported to be associated with endothelial dysfunction [4–8]. Protein-bound uremic toxins, one of nontraditional factors, include PCS and indoxyl sulfate (IS), and have been regarded to be contributed to this pathophysiology [10, 18].

PCS, an endproduct of protein metabolism originating from intestinal tract, is accumulated as renal function declines [19]. From *in vitro* studies, it showed an increased free radical production after exposure of leukocyte to PCS at a uremia concentration [20]. In addition, Meijers et al. reported that PCS could promote endothelial microparticle release, an indicator of endothelial damage [10]. Both of endothelial damage and leukocyte activation are able to contribute to vascular damage [21]. However, the serum concentration of PCS was increased gradually in patients with advanced CKD [22] and could not be effectively removed by standard dialysis [9]. It subsequently will carry clinical toxicity finally. This can be proved by some previous prospective studies, which demonstrated a close relationship between PCS levels and clinical outcomes including infectious event, cardiovascular disease, and overall mortality in a hemodialysis [12–14] and peritoneal dialysis cohort [23].

In this study, we observed that, in pre-ESRD patients, the PCS level was able to predict cardiovascular event during study period. Our results were partially concordant with the findings published by Liabeuf et al., except overall mortality event [22]. There was no significant correlation between PCS levels and overall mortality event in this research. This discrepancy results from lower mortality rate in our patients and reflects the different survival rate of CKD in western and eastern country. Our recent study also indicated that PCS level was not only associated with peripheral artery disease but also a valuable surrogate marker in predicting vascular access dysfunction in patient with hemodialysis [15]. Another previous study revealed that PCS level was correlated with coronary lesions in patients with stable angina and moderate degrees of CKD [24]. These findings specify the accumulation of PCS was closely linked to unfavorable cardiovascular outcomes in CKD population.

However, based on previous reports, the effect of PCS on kidney progression has not been demonstrated. Until a recent basic research conducted by Watanabe et al., indicated PCS was capable of resulting in renal tubular cell damage by inducing oxidative stress by activation of NADPH oxidase [25], a similar mechanism caused by IS [26, 27]. This is the first study to support renal toxicity of PCS. It also can explain the result from our study that PCS level could predict kidney function deterioration. Our finding can be regarded as the extension of results from basic *in vitro* to clinical research. Thus, these evidences indicated that PCS was not only a vascular toxin but also a nephrotoxin. There are limitations in our study. First, this study was performed with only minimum numbers of study patients, and all subjects were enrolled from one medical center. Second, whether attenuation of serum PCS concentration could reduce the risk of cardiovascular event and delay kidney function progression is still unclear.

In conclusion, our study showed higher serum PCS levels were closely associated with cardiovascular event and dialysis event. It provides more evidences about the toxic effect of PCS on clinical outcomes. Further more studies are needed to demonstrate if patient's outcomes could be improved after lowering serum PCS levels in future.

## Figures and Tables

**Table 1:** Baseline characteristics of the study patients.

Variables	All (*n* = 72)
Age (yr)	60.1 ± 9.4
Male (%)	50%
Diabetes mellitus (%)	31.9%
Hypertension (%)	43.1%
CVD (%)	15.3%
SBP (mmHg)	141.5 ± 15.7
DBP (mmHg)	73.3 ± 11.5
CKD stage (%)	
3	34.8%
4	32.4%
5	32.8%
Albumin (g/dL)	4.01 ± 0.4
Hemoglobin (g/L)	10.3 ± 1.4
Hematocrit (%)	31.4 ± 5.6
BUN (mg/dL)	44.1 ± 23.3
Creatinine (mg/dL)	3.8 ± 2.6
eGFR (mL/min)	23.6 ± 15.1
Calcium (mg/dL)	9.1 ± 0.4
Phosphate (mg/dL)	4.5 ± 0.8
Intact-PTH (pg/mL)	132.5 ± 177.1
P-cresyl sulfate (mg/L)	7.7 ± 7.2

Values expressed as mean ± SD or percent. CVD: cardiovascular disease; CKD: chronic kidney disease; SBP: systolic blood pressure; DBP: diastolic blood pressure; eGFR: estimated GFR.

**Table 2:** Clinical biochemistry of CKD patients divided by medium PCS concentration (6 mg/L).

Variables	P-cresyl sulfate ≧6.0 mg/L	P-cresyl sulfate <6.0 mg/L	*P* value
	(*n* = 34)	(*n* = 38)	
CKD stage (%)			
3	10.0%	57.3%	<0.001
4	24.5 %	37.5%	<0.001
5	65.5%	5.2%	<0.001
Albumin (g/dL)	3.9 ± 0.3	4.0 ± 0.4	NS
Hemoglobin (g/L)	9.3 ± 1.7	10.7 ± 1.9	0.01
Hematocrit (%)	27.8 ± 5.7	32.3 ± 5.5	0.01
BUN (mg/dL)	52.0 ± 23.4	37.8 ± 20.8	<0.001
Creatinine (mg/dL)	4.9 ± 2.9	2.8 ± 2.1	<0.001
eGFR (mL/min)	15.8 ± 15.3	30.5 ± 15.4	<0.001
Calcium (mg/dL)	9.1 ± 0.5	9.0 ± 0.4	NS
Phosphate (mg/dL)	4.6 ± 0.7	4.3 ± 0.7	NS
Intact-PTH (pg/mL)	201.1 ± 230	78.5 ± 75.0	<0.001
P-cresyl sulfate (mg/L)	13.4 ± 6.4	2.5 ± 1.8	<0.001

**Table 3:** Univariate and multivariate Cox regression analysis for evaluating the relationship between independent variables and clinical outcomes in CKD patients.

Variables	Cardiovascular event						All-cause mortality			Dialysis					
	Univariate Cox regression analysis			Multivariate Cox regression analysis			Univariate Cox regression analysis			Univariate Cox regression analysis			Multivariate Cox regression analysis		
	HR	95% CI	*P*	HR	95% CI	*P*	HR	95% CI	*P*	HR	95% CI	*P*	HR	95% CI	*P*
Gender (F/M)	0.66	0.24–1.77	NS				0.84	0.12–5.99	NS	0.80	0.29–2.21	NS			
Age (years)	1.08	1.01–1.15	0.01	1.12	1.00–1.25	0.01	1.12	0.95–1.30	NS	0.93	0.89–0.98	<0.01	0.91	0.85–0.96	<0.01
CV/Non-CV	2.92	1.01–8.41	0.04	6.28	1.32–29.71	0.02	0.04	0.00–31.81	NS	0.72	0.16–3.19	NS			
DM/Non-DM	1.80	0.67–4.83	NS				2.19	0.31–15.58	NS	0.52	0.14–1.84	NS			
BUN (mg/dL)	1.03	1.00–1.04	<0.01	0.98	0.95–1.03	NS	0.99	0.94–1.03	NS	1.05	1.02–1.07	<0.01	0.99	0.95–1.03	NS
Cr (mg/dL)	1.11	0.97–1.28	NS				0.89	0.56–1.41	NS	1.51	1.31–1.73	<0.01	1.25	0.74–2.11	NS
eGFR (mL/min)	0.95	0.91–0.99	<0.01	0.99	0.93–1.06	NS	0.98	0.92–1.05	NS	0.80	0.71–0.90	<0.01	0.69	0.47–1.02	NS
Hb (g/dL)	0.85	0.82–1.93	NS				0.79	0.56–1.04	NS	0.85	0.33–2.31	NS			
Hct (%)	0.90	0.33–2.42	NS				3.77	0.39–36.31	NS	0.91	0.82–1.01	NS			
Albumin (g/dL)	0.82	0.25–2.65	NS				0.85	0.06–10.82	NS	0.39	0.15–0.99	0.04	0.03	0.00–0.33	<0.01
Ca (mg/dL)	0.27	0.11–0.65	<0.01	0.54	0.19–1.54	NS	3.29	0.44–24.11	NS	0.41	0.15–1.10	NS			
P (mg/dL)	1.97	1.16–3.33	0.01	1.66	0.70–3.91	NS	0.41	0.08–2.02	NS	2.06	1.15–3.72	0.01	0.93	0.53–1.02	NS
i-PTH (pg/mL)	1.00	1.00-1.00	0.03	1.00	0.99–1.01	NS	1.00	0.99–1.01	NS	1.00	1.00-1.00	<0.01	0.99	0.99-1.00	NS
PCS (mg/L)	1.08	1.02–1.15	<0.01	1.12	1.01–1.21	0.02	1.06	0.94–1.21	NS	1.10	1.02–1.17	<0.01	1.17	1.05–1.30	<0.01

PCS: P-cresyl sulfate, NS: no significance, CI: confidence interval.

## References

[1] Keith DS, Nichols GA, Gullion CM, Brown JB, Smith DH (2004). Longitudinal follow-up and outcomes among a population with chronic kidney disease in a large managed care organization. *Archives of Internal Medicine*.

[2] Sarnak MJ, Levey AS, Schoolwerth AC (2003). Kidney disease as a risk factor for development of cardiovascular disease: a statement from the American Heart Association Councils on Kidney in Cardiovascular Disease, High Blood Pressure Research, Clinical Cardiology, and Epidemiology and Prevention. *Hypertension*.

[3] Go AS, Chertow GM, Fan D (2004). Chronic kidney disease and the risks of death, cardiovascular events, andhospitalization. *The New England Journal of Medicine*.

[4] Sarnak MJ, Coronado BE, Greene T (2002). Cardiovascular disease risk factors in chronic renal insufficiency. *Clinical Nephrology*.

[5] Clarke R, Daly L, Robinson K (1991). Hyperhomocysteinemia: an independent risk factor for vascular disease. *The New England Journal of Medicine*.

[6] Culleton BF, Wilson PW (1998). Cardiovascular disease: risk factors, secular trends, and therapeutic guidelines. *Journal of the American Society of Nephrology*.

[7] Sarnak MJ, Levey AS (2000). Cardiovascular disease and chronic renal disease: a new paradigm. *The American Journal of Kidney Diseases*.

[8] Longenecker JC, Coresh J, Powe NR (2002). Traditional cardiovascular disease risk factors in dialysis patients compared with the general population: the CHOICE study. *Journal of the American Society of Nephrology*.

[9] Krieter DH, Hackl A, Rodriguez A (2010). Protein-bound uraemic toxin removal in haemodialysis and post-dilution haemodiafiltration. *Nephrology Dialysis Transplantation*.

[10] Meijers BK, Van kerckhoven S, Verbeke K (2009). The Uremic Retention Solute p-Cresyl Sulfate and Markers of Endothelial Damage. *The American Journal of Kidney Diseases*.

[11] Dou L, Bertrand E, Cerini C (2004). The uremic solutes p-cresol and indoxyl sulfate inhibit endothelial proliferation and wound repair. *Kidney International*.

[12] Meijers BK, Bammens B, de Moor B, Verbeke K, Vanrenterghem Y, Evenepoel P (2008). Free p-cresol is associated with cardiovascular disease in hemodialysis patients. *Kidney International*.

[13] Lin CJ, Wu CJ, Pan CF, Chen YC, Sun FJ, Chen HH (2010). Serum protein-bound uraemic toxins and clinical outcomes in haemodialysis patients. *Nephrology Dialysis Transplantation*.

[14] Bammens B, Evenepoel P, Keuleers H, Verbeke K, Vanrenterghem Y (2006). Free serum concentrations of the protein-bound retention solute p-cresol predict mortality in hemodialysis patients. *Kidney International*.

[15] Lin CJ, Pan CF, Liu HL (2012). The role of protein-bound uremic toxins on peripheral artery disease and vascular access failure in patients on hemodialysis. *Atherosclerosis*.

[16] Foley RN, Parfrey PS, Sarnak MJ (1998). Epidemiology of cardiovascular disease in chronic renal disease. *Journal of the American Society of Nephrology*.

[17] Halcox JPJ, Schenke WH, Zalos G (2002). Prognostic value of coronary vascular endothelial dysfunction. *Circulation*.

[18] Masai N, Tatebe J, Yoshino G, Morita T (2010). Indoxyl sulfate stimulates monocyte chemoattractant protein-1 expression in human umbilical vein endothelial cells by inducing oxidative stress through activation of the NADPH oxidase-nuclear factor-*κ*B pathway. *Circulation Journal*.

[19] Lin CJ, Chen HH, Pan CF (2011). p-cresylsulfate and indoxyl sulfate level at different stages of chronic kidney disease. *Journal of Clinical Laboratory Analysis*.

[20] Schepers E, Meert N, Glorieux G, Goeman J, van der Eycken J, Vanholder R (2007). P-cresylsulphate, the main in vivo metabolite of p-cresol, activates leucocyte free radical production. *Nephrology Dialysis Transplantation*.

[21] Vanholder R, Argilés A, Baurmeister U (2001). Uremic toxicity: present state of the art. *International Journal of Artificial Organs*.

[22] Liabeuf S, Barreto DV, Barreto FC (2010). Free p-cresylsulphate is a predictor of mortality in patients at different stages of chronic kidney disease. *Nephrology Dialysis Transplantation*.

[23] Lin CJ, Pan CF, Chuang CK (2013). Gastrointestinal-related uremic toxins in peritoneal dialysis: a pilot study with a 5-year follow-up. *Archives of Medical Research*.

[24] Wang CP, Lu LF, Yu TH (2010). Serum levels of total p-cresylsulphate are associated with angiographic coronary atherosclerosis severity in stable angina patients with early stage of renal failure. *Atherosclerosis*.

[25] Watanabe H, Miyamoto Y, Honda D (2013). p-Cresyl sulfate causes renal tubular cell damage by inducing oxidative stress by activation of NADPH oxidase. *Kidney International*.

[26] Tumur Z, Niwa T (2009). Indoxyl sulfate inhibits nitric oxide production and cell viability by inducing oxidative stress in vascular endothelial cells. *The American Journal of Nephrology*.

[27] Yu M, Kim YJ, Kang DH (2011). Indoxyl sulfate-induced endothelial dysfunction in patients with chronic kidney disease via an induction of oxidative stress. *Clinical Journal of the American Society of Nephrology*.

